# The impact of the quality of care and other factors on progression of chronic kidney disease in Thai patients with Type 2 Diabetes Mellitus: A nationwide cohort study

**DOI:** 10.1371/journal.pone.0180977

**Published:** 2017-07-28

**Authors:** Paithoon Sonthon, Supannee Promthet, Siribha Changsirikulchai, Ram Rangsin, Bandit Thinkhamrop, Suthee Rattanamongkolgul, Cameron P. Hurst

**Affiliations:** 1 Doctor of Public Health Program, Faculty of Public Health, Khon Kaen University, Khon Kaen, Thailand; 2 Phetchabun Provincial Public Health Office, Ministry of Public Health, Phetchabun, Thailand; 3 Faculty of Public Health, Khon Kaen University, Khon Kaen, Thailand; 4 Faculty of Medicine, Srinakharinwirot University, Nakhon Nayok, Thailand; 5 Department of Military and Community Medicine, Phramongkutklao College of Medicine, Bangkok, Thailand; 6 Center of Excellence in Biostatistics, Faculty of Medicine, Chulalongkorn University, Bangkok, Thailand; Shanghai Diabetes Institute, CHINA

## Abstract

**Objective:**

The present study investigates the impact of quality of care (QoC) and other factors on chronic kidney disease (CKD) stage progression among Type 2 Diabetes Mellitus (T2DM) patients.

**Methods:**

This study employed a retrospective cohort from a nationwide Diabetes and Hypertension study involving 595 Thai hospitals. T2DM patients who were observed at least 2 times in the 3 years follow-up (between 2011–2013) were included in our study. Ordinal logistic mixed effect regression modeling was used to investigate the association between the QoC and other factors with CKD stage progression.

**Results:**

After adjusting for covariates, we found that the achievement of the HbA1c clinical targets (≤7%) was the only QoC indicator protective against the CKD stage progression (adjusted OR = 0.76; 95%CI = 0.59–0.98; p<0.05). In terms of other covariates, age, occupation, type of health insurance, region of residence, HDL-C, triglyceride, hypertension and insulin sensitizer were also strongly associated with CKD stage progression.

**Conclusions:**

This cohort study demonstrates the achievement of the HbA1c clinical target (≤7%) is the only QoC indicator protective against progression of CKD stage. Neither of the other clinical targets (BP and LDL-C) nor any process of care targets could be shown to be associated with CKD stage progression. Therefore, close monitoring of blood sugar control is important to slow CKD progression, but long-term prospective cohorts are needed to gain better insights into the impact of QoC indicators on CKD progression.

## Introduction

Chronic kidney disease (CKD) is a major problem in both developing and developed countries and is associated with a substantial burden in terms of mortality, morbidity, and health care costs [1–3]. Type 2 diabetes mellitus (T2DM) is the leading cause of CKD worldwide and the prevalence of CKD in T2DM patients is 43.5% in the US [4], 38% in Belgium [5], 34.7% in Finland [6] and 22.9% in the Mediterranean area [7]. Furthermore, CKD patients have 2 to 4 times the risk of cardiovascular disease [8], and 3 times the risk of mortality relative to T2DM patients without CKD [9]. Also, quality of life has been shown to be negatively associated with CKD [10].

The quality of care (QoC) protocol for T2DM is well established in improving health outcomes, slowing progression and prolonging the onset of T2DM complication including CKD [11]. Several studies have demonstrated that both process of care and clinical targets are important indicators for assessing and monitoring QoC in diabetes [12]. The process of care refers to the proportion of patients with T2DM who receive core examinations for diabetes in any given 12 months period, based on Standard of Medical Care-2014 [13]. The clinical targets of care refers to successful achievement of the ABC goal where A is glycated hemoglobin control (HbA1c)≤7%, B is blood pressure control(BP)≤130/80mmhg, and C is low-density lipoprotein cholesterol control (LDL-C)<100mg/d. The ABC clinical targets are widely used for both diabetes [13] and CKD [14] management.

Few studies have investigated the impact the QoC on the prevalence or progression of CKD in T2DM patients. Of those that have been done, most studies have been conducted in Western populations. Furthermore, CKD progression is typically measured using a binary form of progression versus non-progression [15] and to the best of our knowledge no previous study has considered CKD stage progression (as defined by 2012KDIGO) [14]. The present study investigates how the QoC impact on CKD stage progression using a nationwide retrospective cohort in Thailand.

## Materials and methods

### Study design and populations

Data for this study were obtained from a nationwide, multicenter survey involving three consecutive yearly cross-sectional samples of both T2DM and Hypertension outpatients in Thailand. The full study is titled: ‘‘An assessment of quality of care among patients diagnosed with type 2 diabetes and hypertension visiting hospitals in care of Ministry of Public Health and Bangkok Metropolitan Administration in Thailand” This project is administered by the Medical Research Network of the Consortium of Thai Medical Schools (MedResNet), Thailand, under the sponsorship of the Thai National Health Security Office. Patients and hospitals were sampled using proportion-to-size stratified cluster sampling of T2DM and hypertension outpatients from 595 participating hospitals across Thailand between April to June for the years 2011 to 2013. All patient information was retrospectively collected via medical records.

**Fig 1:**
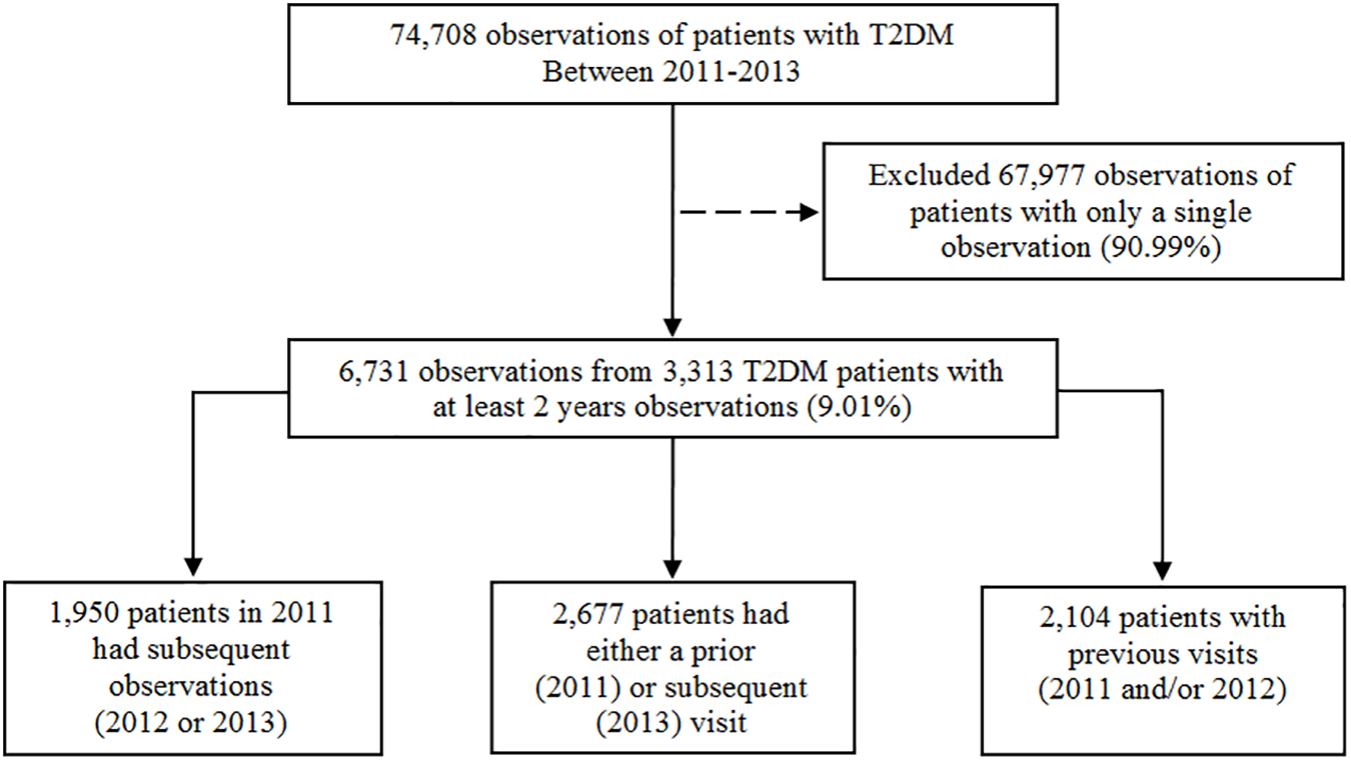
Study flow.

The Diabetes and Hypertension dataset (DMHT), study protocols and case report forms are archived at the website http://www.damus.in.th/damus/. Patients were eligible for inclusion if they had received care and treatment for T2DM or hypertension in a participating hospital for at least 12 months. For the present study, only patients assessed at least 2 of the 3 years between the period 2011–2013 were included. That is patients with repeated observations during the study period. Patients who did not have complete information on the outcome or study effects (QoC) were excluded. Fig 1 provides the study flow.

### Measurements

The outcome of this study is progression of CKD stage based on prognostic stages as represented by 2012KDIGO [14] where eGFR stages are integrated with albuminuria stages and subsequently divided into 4 stages: low risk; moderately increased risk; high risk and very high risk. The progression of CKD is represented as a change of CKD stage over the course of our study period.

The study effect in the present study is represented by the QoC which included two components: clinical targets of care and processes of care. Clinical targets of care were defined as the achievement of (A) HbA1c≤7%, (B)BP≤130/80 mmHg, and (C) LDL-C<100mg/dL(Commonly referred to as the ABC of diabetes). Processes of care were represented as the receipt of core diabetes clinical examinations being completed every 12 months and include Foot examination (F exam), HbA1c examination (A exam), Cholesterol examination(C exam), and Eye examination (E exam) (collectively referred to as FACE) [16].

Other covariates included as potential predictors and/or confounders in this study were gender, age, occupation, religion, health insurances scheme, smoking history, body mass index, duration of diabetes, hospital type (community, provincial and regional), region of residence, total cholesterol, HDL-cholesterol, triglyceride, ACEI or ARBs, anticoagulants, diabetes medication, hypertension, neuropathy and retinopathy.

### Statistical analysis

Continuous variables were described using means and standard deviations, and categorical variables were summarized using counts and percentages. As the outcome of the present study, CKD stage, is represented on an ordinal scale and measured on at least two occasions for each patient, ordinal logistic mixed effect regression was used to model the ordinal outcome CKD stage (low risk, moderately increased risk, high risk, and very high risk). The study effects, the ABC clinical targets, and the process of care targets were the focus in their respective models. The selection of other risk factors and/or confounders to be included in multivariable models was based on a p<0.25 in the bivariate models. Unadjusted or adjusted Odd ratios with 95% confident interval (CI) and p-values were used to examine associations between the QoC and other covariates with CKD stage progression. To assess the impact of missing values (bias vs. loss of precision), the odd ratios between the complete case and available case data analyses were compared. Available cases were patients that were bivariate complete for any given bivariate analysis (they only needed non-missing values for the two variables being considered in that bivariate analysis, but could be missing for other variables), whereas complete cases represented patients with non-missing values for all of the variables considered in the full multivariable model. All analysis were conducted using the R statistical language (v3.2.4) [17] and the ordinal mixed model was performed using the ordinal R library [18]. A significance level of 0.05 was used throughout all analysis.

### Ethical approval

This study was approved by the Ethics Committee for Human Research Khon Kaen University, Thailand (HE582363) and written informed consent was obtained from all patients prior to inclusion in the study.

## Results

Of the total number of patients in the Thai DMHT study, 3,313(9.01%) patients had 2 or more observations (collectively, 6,731 observations) and were subsequently included in the present study (Fig 1). To assess whether our sample (patients with at least two observations) is representative of the full T2DM dataset (all T2DM patients), we compared patients with repeated observation with the 67,977 patients only observed once (Table 1). For the most part, patients with a single observations were similar to those with repeated observations. For example, there was less than 1 percent fewer patients with repeated observations who achieved the blood pressure clinical target (Table 2). However, there was some difference (3.26%) in the achievement of HbA1c control between patients with a single observation, and those with repeated observations.

**Table 1:** Baseline of characteristics of patients with the two or more and with only a single observation.

Variables	Single observation	≥Two observations	%/mean
	(67,977 patients)	(3,313 patients)	difference
Female gender, n (%)	47,148(69.36)	2,351(70.96)	1.60
Age, years, mean(sd.)	60.20(10.68)	59.69(10.35)	0.51
<40, n (%)	1,694(2.49)	95(2.87)	
40 to 59, n (%)	30,691(45.16)	1,526(46.06)	
60 to 79, n (%)	33,341(49.06)	1,606(48.48)	
≥80, n (%)	2,239(3.29)	86(2.60)	
	Min = 20,Max = 98	Min = 26, Max = 92	
Duration of diabetes, years, mean(sd.)	6.78(4.66)	6.81(4.63)	-0.03
Occupation, n (%)			
Government	3,866(5.96)	188(5.96)	0.00
Homemaker	15,154(23.37)	699(22.16)	-1.21
Other	5,315(8.20)	273(8.66)	0.46
Agriculture	28,630(44.15)	1,458(46.22)	2.07
Labor	11878(18.32)	536(16.99)	-1.33
Religion, Buddhism, n (%)	69,972(96.27)	2,955(96.22)	-0.05
Health insurances scheme, n (%)			
Universal coverage	52,951(78.23)	2,545(77.21)	-1.02
Government	11,726(17.32)	583(17.69)	0.37
Social security	2,596(3.83)	152(4.61)	0.78
Other	412(0.60)	16(0.49)	-0.11
Hospital type, n (%)			
Regional hospital	8,689(13.63)	472(15.39)	1.76
Provincial hospital	14,601(22.90)	598(19.49)	-3.41
Community hospital	40,470(63.47)	1,997(65.11)	1.64
Region of residence, n (%)			
Central	22,326(32.84)	1,080(32.60)	-0.24
North	13,966(20.54)	673(20.31)	-0.23
South	8,090(11.90)	401(12.10)	0.20
North-Eastern	23,595(34.71)	1,159(34.98)	0.27
BMI (kg/m2), n (%)			
<18.50	2,222(3.49)	97(3.14)	-0.35
18.5 to 22.9	16,347(25.68)	755(24.44)	-1.24
23.0 to 27.4	26,891(42.24)	1,335(43.21)	0.97
≥ 27.5	18,202(28.59)	902(29.20)	0.61
Smoking history, n (%)			
no	52,202(89.05)	2,488(90.40)	1.35
previous	2,662(4.54)	94(3.41)	1.13
yes	3,754(6.40)	170(6.18)	-0.22
Total cholesterol(mg/dL), mean(sd.)	187.73(45.13)	187.83(45.14)	0.1
HDL-C(mg/dL), mean(sd.)	45.89(13.16)	45.09(11.89)	-0.8
LDL-C(mg/dL), mean(sd.)	109.33(37.25)	108.96(36.78)	-0.37
Triglyceride(mg.dL), mean(sd.)	173.03(96.41)	175.00(93.94)	1.97
HbA1c(%), mean(sd.)	8.03(2.13)	8.14(2.04)	0.11
SBP(mmHg), mean(sd.)	128.72(16.37)	129.43(17.03)	0.71
DBP(mmHg), mean(sd.)	74.23(10.53)	74.51(10.84)	0.28
Hypertension, n (%)	47,864(70.41)	2,277(68.73)	-1.68
Neuropathy (>12months), n (%)	653(0.96)	37(1.11)	0.15
Retinopathy (>12months), n (%)	1,460(2.14)	103(3.11)	0.97

BP: blood pressure; BMI: body mass index; HbA1c: glycosylated hemoglobin; HDL-C: high-density lipoprotein cholesterol; LDL-C: low-density lipoprotein cholesterol; sd.: standard deviations; SBP: Systolic Blood Pressure; DBP: Diastolic Blood Pressure

**Table 2:** Medication and quality of care of patients with the two or more and with only a single observation.

Variables	Single observation	≥Two observations	%/mean
	(67,977 patients)	(3,313 patients)	difference
ACEI or ARBs, n (%)			
No	29,653(44.77)	1,565(48.53)	3.76
Previous	1,305(1.97)	2(0.06)	-1.91
Yes	35,272(53.26)	1,658(51.41)	-1.85
Diabetes medication, n (%)			
No medication	1861(2.79)	70(2.15)	-0.64
OHAs	50,728(76.03)	2,478(76.18)	0.15
Insulin sensitizer	6,073(9.10)	251(7.71)	-1.39
Both of oral & insulin	8,055(12.07)	454(13.96)	1.89
Anticoagulation, n (%)	39,966(60.08)	1,987(61.71)	1.63
Proportion of achieved ABC, mean(sd.)	0.46(0.33)	0.45(0.33)	-0.1
**Quality of Care**			
**Clinical targets of care**, n (%)			
HbA1c target, Yes	19,135(37.19)	837(33.93)	-3.26
Blood pressure target, Yes	36,399(54.64)	1,740(53.65)	-0.99
LDL-C target, Yes	24,647(43.37)	1,174(43.11)	-0.26
All ABC target, Yes	4594(6.87)	206(6.33)	-0.54
**Process of care**, n (%)			
Foot exam, Yes	43,710(64.30)	2,175(65.65)	1.35
HbA1c exam, Yes	51,484(75.73)	2,467(74.46)	-1.27
Cholesterol exam, Yes	56,906(83.71)	2,724(82.22)	-1.49
Eye exam, Yes	35,420(52.11)	1,782(53.79)	1.68

ACEI: angiotensin-converting-enzyme inhibitor; ARB: angiotensin receptor blockers; HbA1c: glycosylated hemoglobin; LDL-C: low-density lipoprotein cholesterol; OHAs: oral hyperglycemic agent; sd.: standard deviations

### Baseline characteristics and quality of care

Baseline characteristics of patients with CKD are listed in Table 1. A majority of patients were female (70.96%), and the mean age and duration of DM in patients were 59.69 years (sd. = 10.35) and 6.81 years (sd. = 4.63), respectively. Most patients had universal health coverage (77.21%), and a majority received their assessment in a community hospital setting (65.11%). Over 70% of patients were overweight or obese (≥23.0), and patients who had never smoked represented 90.40% of the sample. More than 75% of patients were receiving oral hyperglycemic agents (OHAs), and approximately one-half of patients were being treated with anti-hypertensive agents (Table 2). Indeed, a hypertension morbidity was very high (68.73%). In terms of DM complications, 1.11% and 3.11% had neuropathy and retinopathy, respectively (Table 1).

With regards to the percentage of CKD patients achieving the DM QoC targets, 53.65% achieved the blood pressure clinical target, followed by 43.11% achieved the LDL-C clinical target, and 33.93% achieved the HbA1c clinical target. For the process of care, 82.22% had Cholesterol examinations, followed by 74.46% receiving HbA1c examinations, 65.65% having Foot examinations, and 53.79% receiving Eye examinations (Table 2).

**Fig 2:**
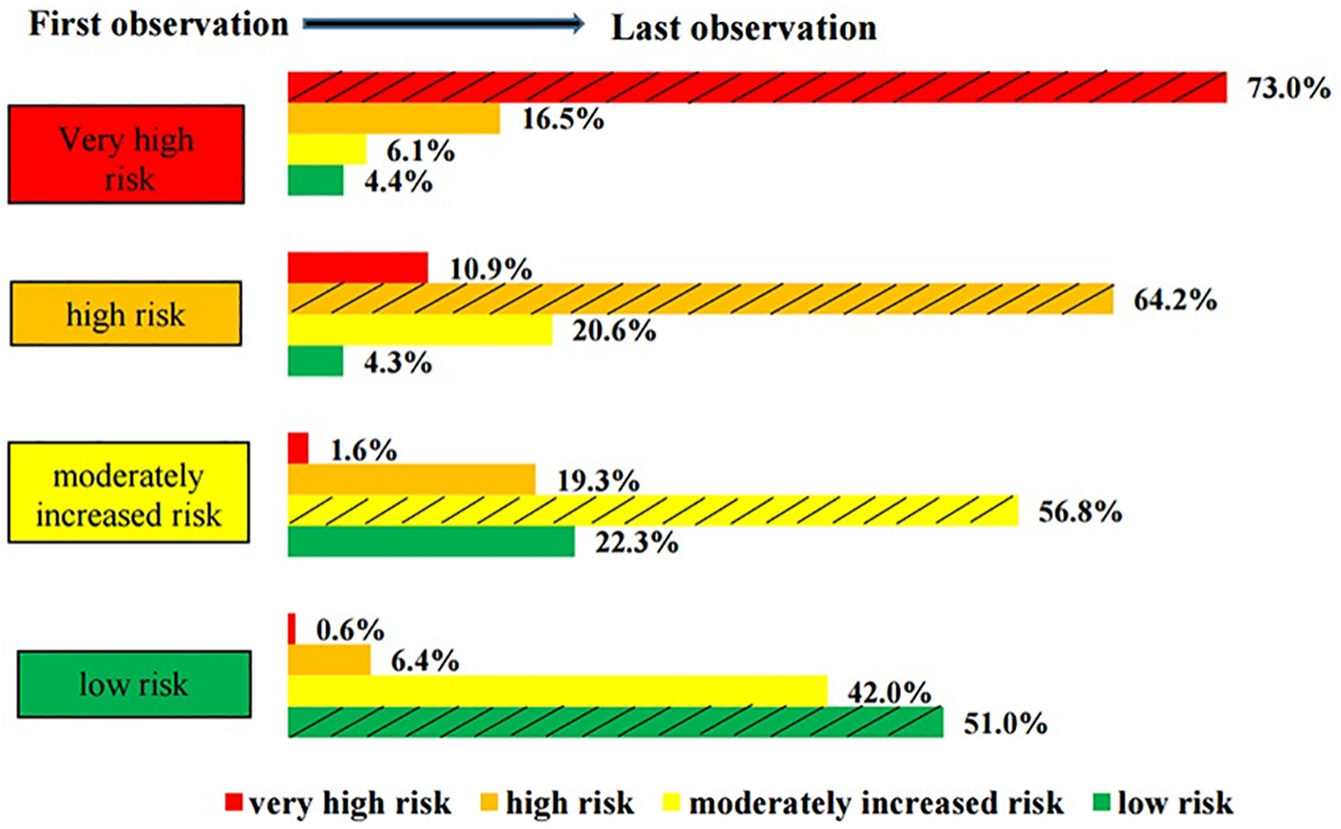
CKD stage progression from the first observation to the last observation. **(1,630 patients).** Shaded bars represent stable patients whereas those below the shaded bars represent improved CKD stage, and those above the bars represent patients whose CKD stage progressed.

The CKD stage progression is illustrated in Fig 2. Review of this figure suggests that a large majority of patients remained stable over the course of the study (shaded bars). It is interesting to note that for the intermediate categories, moderately increased risk, the proportion of patients with CKD progression was approximately the same as the proportion whose condition improved stage. Also, a very large proportion of patients starting in the low risk group progressed to a higher CKD stage during our study period.

### Clinical targets and progression of CKD

The unadjusted and adjusted odd ratios representing the association between the clinical targets of care and progression of CKD are shown in Table 3. The bivariate analysis suggests all three clinical targets are associated with the progression of CKD stage and all appear to be protective against CKD progression. The odds of progression for someone who achieved HbA1c is 33% (OR = 0.66; p<0.01) less than someone who did not achieve this target. For the BP target, those that achieved this target had odds of progression 25% (OR = 0.75; p<0.05) less than those who did not achieve the BP target. Finally, those achieving LDL-C target, had 31% (OR = 0.69; p<0.05) lower odds of CKD progression relative to those not achieving cholesterol control. However, after adjusting for others covariate, only the HbA1c clinical target remained significantly protective against CKD stage progression (adjusted OR = 0.76; 95%CI = 0.59–0.98; p<0.05).

**Table 3:** Association between clinical targets of care and progression of CKD.

Factors	Available case		Complete case		
	(6,731obs.)		(2,588 obs. from 1,763 patients)		
	n_patients_	Unadjusted	Unadjusted	Adjusted	95%CI
		OR	OR	OR	
HbA1c target, Yes	2,467	0.72**	0.66**	0.76*	0.59–0.98
Blood pressure target, Yes	3,243	0.81*	0.75*	0.94	0.75–1.19
LDL-C target, Yes	2,723	0.80*	0.69**	0.88	0.65–1.19
**Covariates**					
Gender, Female	3,313	1.09	1.30	–	–
Age (in10years)	3,313	3.19***	3.44***	2.70***	2.27–3.22
Duration of Diabetes (in5years)	3,138	2.65***	2.87***	–	–
Occupation, Government(ref.)	3,154	χ2 = 79.84***	χ2 = 71.27***	χ2 = 16.11**	
Homemaker		5.09***	10.31***	3.03***	1.63–5.63
Other		1.89	2.47*	1.99	0.97–4.10
Agriculture		3.75***	4.91***	2.87***	1.59–5.19
Labor		1.30	1.88	2.15*	1.14–4.06
Religion, other	3,071	1.14	0.97	–	–
Health insurances, UC (ref.)	3,296	χ2 = 20.73***	χ2 = 13.41**	–	
Government		0.84	0.80	–	–
Social security		0.25 ***	0.22***	–	–
Other		2.59	1.10	–	–
Hospital type, Region(ref.)	3,067	χ2 = 25.13***	χ2 = 11.30**	–	
Provincial hospital		2.54 ***	2.19**	–	–
Community hospital		2.49 ***	2.33**	–	–
Region, Central(ref.)	3,313	χ2 = 43.74***	χ2 = 19.37***	χ2 = 8.00*	
North		2.11***	2.10**	1.30	0.87–1.94
South		1.57 *	1.34	1.22	0.78–1.90
North-Eastern		2.94 ***	2.68 ***	1.71**	1.15–2.53
BMI(kg/m2), 18.5to22.9(ref.) ((3089	3,089	χ2 = 10.23*	χ2 = 7.04	–	
<18.50		1.10	1.61	–	–
23.0 to 27.4		0.65**	0.69*	–	–
≥ 27.5		0.70*	0.65	–	–
Smoking History, No(ref.)	2,752	χ2 = 1.08	χ2 = 0.97	–	
Previous		0.89	0.72	–	–
Yes		1.22	1.12		–
Total cholesterol(mg/dL)	2,716	1.004 ***	1.005**	1.003	0.999–1.006
HDL-C(mg/dL)	2,532	0.98 ***	0.97 ***	0.98 ***	0.97–0.99
Triglyceride(mg/dL)	2,798	1.002***	1.003 ***	1.002**	1.000–1.003
Hypertension, Yes	3,313	2.31***	2.67***	1.67**	1.22–2.28
Neuropathy(>12months), Yes	3,313	5.13 ***	6.02***	3.29*	1.31–8.25
Retinopathy(>12months), Yes	3,313	2.43**	1.32	–	–
ACEI or ARBs No(ref.)	3,225	χ2 = 16.56***	χ2 = 15.62***	χ2 = 6.32*	
Previous		3.27 ***	5.33***	2.38*	1.13–5.00
Yes		1.00	1.20	0.94	0.72–1.24
Diabetes medication, No(ref.)	3,253	χ2 = 289.51***	χ2 = 189.13***	χ2 = 162.48***			
OHAs		0.30 **	0.90	1.17	0.46–2.94
Insulin sensitizer		7.70 ***	29.60***	22.63***	8.12–63.08
Both of oral & insulin		0.58	1.62	2.00	0.75–5.32
Anticoagulation, yes	3,220	1.54***	1.67**	–	–

ACEI: angiotensin-converting-enzyme inhibitor; ARB: angiotensin receptor blockers; BMI: body mass index

CI: confidence interval; DM: diabetes mellitus; HbA1c: glycosylated hemoglobin; HDL-C: high-density lipoprotein cholesterol; LDL-C: low-density lipoprotein cholesterol; OHAs: oral hyperglycemic agents; OR: odds ratio

ref.: reference; obs: observations; UC: universal coverage

*** p<0.001

** p<0.01

* p<0.05.

Table 3 also shows the association of other predictors with CKD progression. Older age (per 10 years) was a strong predictor of progression of CKD stage (adjusted OR = 2.70; 95%CI = 2.27–3.22; p<0.001). Patients living in the North-Eastern region had a higher risk CKD stage progression relative to patients residing in the Central region (adjusted OR = 1.71; 95%CI = 1.15–2.53; p<0.01). In terms of occupation, homemakers (adjusted OR = 3.03; 95%CI = 1.63–5.63; P<0.001) and agriculture workers (adjusted OR = 2.87; 95%CI = 1.59–5.19; p<0.001) had a significantly higher risk of CKD stage progression relative to government workers. There were many other risk factors associated with CKD stage progression (Table 3). In terms of comorbidities, we found patients with hypertension had a higher risk of CKD stage progression (adjusted OR = 1.67; 95%CI: 1.22–2.28; p<0.01). With regards to treatment, insulin sensitizer (adjusted OR = 22.63; 95%CI = 8.12–63.08; p<0.001) and previous ACE inhibitor or angiotensin receptor blockers (adjusted OR = 2.38; 95%CI = 1.13–5.00; p<0.05) were both strongly associated with CKD stage progression.

### Process of care and progression of CKD

The unadjusted and adjusted odd ratios showing the association between the process of care and progression of CKD are given in Table 4. Regardless of whether they were adjusted (or not) for other patients characteristics, most process of care indicators were not associated with CKD stage progression. However, both crude estimates (available case and complete case) suggest eye examination was associated with CKD stage progression (available case OR = 1.22; p<0.05, complete case OR = 1.32; p<0.05). However, when we adjusted for other covariates, we could not demonstrate eye examination, nor any other process of care indicator, was associated with CKD stage progression.

**Table 4:** Association between process of care and progression of CKD.

Factors	Available case	Complete case		
	(6,731obs.)	(2,938 obs. from 1,936 patients)		
	n_patients_	Unadjusted	Unadjusted	Adjusted	95%CI
		OR	OR	OR	
**Process of Care**					
Foot Exam, Yes	3,313	1.22	1.19	0.98	0.76–1.27
HbA1c Exam, Yes	3,313	1.03	0.75	0.85	0.60–1.19
Cholesterol Exam, Yes	3,313	0.85	2.23	2.05	0.73–5.76
Eye Exam, Yes	3,313	1.22*	1.32*	1.22	0.98–1.53
**Covariates**					
Gender, Female	3,313	1.09	1.27	1.39*	1.05–1.84
Age (in10years)	3,313	3.19***	3.379***	2.939***	2.49–3.44
Duration of Diabetes(in5years)	3,138	2.659***	2.869***	–	–
Occupation, Government(ref.)	3,154	χ2 = 79.84***	χ2 = 70.81**	–	
Housekeeper		5.09***	7.85***	–	–
Other		1.89	2.12*	–	–
Agriculture		3.75***	4.08***	–	–
Labor		1.30	1.60	–	–
Religion, Other	3,071	1.14	1.16	1.60	0.73–3.13
Health insurances, UC(ref.)	3,296	χ2 = 20.73***	χ2 = 12.75**	χ2 = 10.57*
Government		0.84	0.76	0.60 **	0.43–0.84
Social security		0.25 ***	0.25 ***	1.22	0.62–2.37
Other		2.58	1.04	0.43	0.06–3.31
Hospital type, Region(ref.)	3,067	χ2 = 25.13***	χ2 = 8.92*	χ2 = 2.90
Provincial hospital		2.54 ***	1.97*	1.47	0.92–2.33
Community hospital		2.49 ***	2.16**	1.41	0.91–2.17
Region, Central(ref.)	3,313	χ2 = 43.74***	χ2 = 21.78***	χ2 = 17.06***
North		2.11***	2.05**	1.41	0.97–2.06
South		1.57 *	1.45	1.30	0.85–2.00
North-Eastern		2.94 ***	2.65***	2.01***	1.42–2.86
BMI(kg/m2), 18.5to22.9(ref.)	3,089	χ2 = 10.23*	χ2 = 10.01*	χ2 = 6.48
<18.50		1.10	1.17	1.24	0.64–2.40
23.0 to 27.4		0.65**	0.61***	0.74*	0.56–0.98
≥ 27.5		0.70*	0.66***	0.94	0.68–1.30
Smoking History, No(ref.)	2,752	χ2 = 1.08	χ2 = -0.028	–
Previous		0.89	0.90	–	–
Yes		1.22	1.00	–	–
Total cholesterol(mg/dL)	2,716	1.004 ***	1.004**	1.003	1.000–1.006
HDL-C(mg/dL)	2,532	0.98 ***	0.97***	0.98***	0.97–0.99
Triglyceride(mg/dL)	2,798	1.002***	1.003***	1.002***	1.001–1.004
Hypertension, Yes	3,313	2.31***	2.49***	1.61**	1.20–2.16
Neuropathy(>12months), Yes	3,313	5.13 ***	5.24***	3.45**	1.48–8.03
Retinopathy(>12months), Yes	3,313	2.43**	1.40	–	–
ACEI or ARBs, No(ref.)	3,225	χ2 = 16.56***	χ2 = 16.67***	χ2 = 7.12*
Previous		3.27 ***	4.48***	2.14*	1.10–4.15
Yes		1.00	1.09	0.90	0.70–1.16
Diabetes medication, No(ref.)	3,253	χ2 = 289.51***	χ2 = 188.28***	χ2 = 174.61***
OHAs		0.30 **	0.87***	1.24	0.54–2.85
Insulin sensitizer		7.70 ***	25.40***	21.33***	8.52–53.42
Both of oral & insulin		0.58	1.47*	2.10	0.87–5.05
Anticoagulation, yes	3,220	1.54***	1.55***	–	–
Proportion of achieved ABC	3,255	0.51***	0.40***	0.65*	0.44–0.97

ACEI: angiotensin-converting-enzyme inhibitor; ARB: angiotensin receptor blockers; BMI: body mass index

CI: confidence interval; HbA1c: glycosylated hemoglobin; HDL-C: high-density lipoprotein cholesterol; LDL-C: low-density lipoprotein cholesterol; OHAs: oral hyperglycemic agents; OR: odds ratio

ref.: reference; obs: observations; UC: universal coverage

*** p<0.001

** p<0.01

* p<0.05.

In terms of the other predictors, the level and significance of associations were similar to those obtained in the clinical targets model (Table 3). However, there was some notable difference in the magnitude and significance of some of the risk factors between the process of care and clinical targets model. Specifically, occupation was significant for the clinical targets model, but not for the process of care model, whereas this situation was reversed for the type of the health insurance which was significant in process of care model, but not clinical targets model (Tables 3 and 4).

## Discussion

CKD represents a major problem as it is associated with a substantial burden in term of mortality [1], morbidity [2] and health care costs [3]. The prevalence of CKD among T2DM patients is increasing rapidly worldwide [4], especially in UMIC (upper middle-income countries) like Thailand [19] and improving the diabetes quality of care in T2DM patients is a strategy to attenuate the CKD problem [11,20]. Our review of the CKD literature found no previous study that has considered CKD stage progression in the Asian population. Furthermore, studies that have considered progression in the other populations do not consider progression through CKD stages as defined by 2012KDIGO. Instead, these studies have focused on a dichotomized measure of progression (progress/ no progress). To the best of our knowledge, our study represents the first study, in any population, to model progression through the 2012KDIGO stages.

### The impact of the QoC on progression of CKD

Our result demonstrates that even after adjusting for other covariates, achieving the HbA1c clinical target (≤7%) is protective against the progression of CKD stage progression. Those that achieved the HbA1c clinical target had a substantially low risk of progressing to the next CKD stage. This result is in line with a meta-analysis review [21] and several cohort studies [22–27] that confirmed higher HbA1c was the risk factor of a decline in eGFR, development of CKD, ESRD and all-cause mortality in patients with T2DM. However, our result contrasts with those of a retrospective cohort study in a single center Vietnamese study involving 450 T2DM patients who did not have renal insufficiency at baseline. In this Vietnamese study, HbA1c control could not be shown to be associated with the development of CKD [20]. However, this could be due to the relatively small sample size of this study, or to the fact that it was a single center study and potentially not representatively of the Vietnamese T2DM population.

Neither of the other clinical targets we considered, BP and LDL-C, could be shown to be associated with progression of CKD stage. This is in line with the results of some previous work. For example, a Norwegian cohort study in the general population (without diabetes, CKD or cardiovascular disease) found that elevated BP was not associated with eGFR decline [28]. However, other studies have shown SBP target (≤130mmHg) to be significantly associated with slowing the rate of decline eGFR in T2DM patients [20,23]. Moreover, our findings contrast with previous studies which indicate that SBP (>130mmHg) is a risk factor for the incidence of albuminuria and renal function in T2DM patients [29,30]. Interestingly, although achievement of BP target could not be shown to be associated with CKD stage progression, we demonstrate a hypertension co-morbidity remains a strong independent risk factor for CKD stage progression. In terms of the LDL-C clinical target, our results are similar to a national cross-sectional study in Italy which found that achievement of the LDL-C target (<100mg/dL) could not be shown to be associated with CKD in patients with T2DM [23].

Our study suggests that process of care indicators such as Foot examinations, HbA1c examinations, Cholesterol examinations, and Eye examinations are not strongly associated with progression of CKD stage. This is in contrast to a study in US diabetes patients which indicated that patients who received the three processes of care, testing HbA1c, lipids, and microalbuminuria were less likely to experience the onset of renal disease, relative to than those who did not receive all three checks [15].

### Other factors associated with CKD progression

Our study demonstrates that some socio-demographic factors were strongly associated with CKD stage progression. For example age, occupation, type of health insurance and region of residence were all strongly associated with CKD stage progression. This result is similar to previous studies which show that advancing age was significantly associated with CKD in T2DM patients [31,32]. Our study found that homemakers and agriculture workers had a substantially higher risk of CKD stage progression relative to government workers. Moreover, our result shows that patients with government health insurance were substantially less likely to experience progression of CKD stage (adjusted OR = 0.60; 95%CI = 0.43–0.84; p<0.01). This result in line with a previous study which indicated that lower socio-economic status was a risk factor for CKD progression [33]. In terms of province of residence, our results are similar to previous studies in Thai adult which reported that CKD was most prevalent in the North-Eastern of Thailand compared with the Central region [19,34]. This result could be due to North-Eastern Thais having a diet richer in glutinous rice relative to other regions, likely to result in poorer HbA1c control [35]. It is important to note, not only is CKD more prevalent in North-Eastern Thailand but so is T2DM [36].

We also found several clinical and co-morbidity factors to be associated with CKD stage progression. In particular, HDL-C, triglyceride, hypertension were all associated with progression. Other studies have demonstrated that HDL-C and triglyceride were associated with an incidence of CKD [37,38], but our study is the first show HDL-C and triglyceride associated with CKD stage progression. In terms of hypertension comorbidity, our findings are in line with those of studies in Western [31] and other Asian populations [39] which identified hypertension is significantly associated with CKD in T2DM patients.

We also found that T2DM treatment with insulin sensitizer was strongly associated with CKD stage progression with patients on this treatment having over 20 times the odds of progression of CKD stage, something that has been demonstrating elsewhere [5]. Indeed, the protocol of diabetes patient care in Thailand recommends the prescription of insulin sensitizer in patients with poor blood sugar control and a high albuminuria [40]. However, it is important to note that insulin itself is unlikely to be a risk factor for progression, but rather insulin treatment is highly associated with advanced T2DM.

There were some limitations in our study. First, this study followed-up patients for three years, an observation period unlikely to capture patents full CKD experience. Second, this study was not designed as a prospective cohort study. It is based on a dataset that randomly sampled a sizable proportion of Thailand T2DM population three years in a row. Our cohort members were those who were captured at least twice by chance. Third, our data were obtained by chart reviews and some importance lifestyle variables that may contribute to CKD stage progression were not recorded in this dataset. For example, physical activity, alcohol consumption, sodium intake and other dietary measures were not measured in our study. This retrospective data collection also led to some data quality issues including many missing values.

Our study also had some strengths. First, progression of CKD stage was based on both Albuminuria and eGFR. Albuminuria, in particular, has been identified as having higher sensitivity to progression than a change in eGFR alone [14]. Second, our study used data from a large and representative multicenter dataset of Thai T2DM patients covering all provinces and health care settings across Thailand. Indeed, 595 hospitals were sampled in our study. Finally, studies of CKD progression in the past have represented CKD progression using a simplistic dichotomized indicator of progress/ no progress. Our study is the first in the world to consider CKD stage (as defined by 2012KDIGO) as an ordinal outcome and we modeled this variable appropriately.

## Conclusions

This cohort study demonstrates the achievement of the HbA1c clinical target (≤7%) was the only clinical target protective against progression of CKD stage. Neither of the other clinical targets, BP and LDL-C, could be shown to be associated with progression of CKD stage. Furthermore, we could not demonstrate that processes of care indicators to be associated with progression of CKD stage. Our study demonstrates the HbA1c target achievement is a strong indicator of CKD stage progression, and CKD patients should be closely monitoring for blood sugar control. Furthermore, staging of CKD should include Albuminuria along with eGFR. Further study of long-term, prospectively-collected cohorts to gain better into the impact of QoC indicators on CKD progression.
